# GmbZIP4a/b Positively Regulate Nodule Number by Affecting Cytokinin Biosynthesis in *Glycine max*

**DOI:** 10.3390/ijms252413311

**Published:** 2024-12-11

**Authors:** Yongjie Meng, Nan Wang, Xin Wang, Zhimin Qiu, Huaqin Kuang, Yuefeng Guan

**Affiliations:** 1 Guangdong Provincial Key Laboratory of Plant Adaptation and Molecular Design, Innovative Center of Molecular Genetics and Evolution, School of Life Sciences, Guangzhou University, Guangzhou 510006, China; meng-yj@gzhu.edu.cn (Y.M.); wangxin@gzhu.edu.cn (X.W.);; 2 School of Life Sciences, Inner Mongolia University, Hohhot 010021, China; wn745970989@163.com; 3 Zhejiang Institute of Subtropical Crops, Zhejiang Academy of Agricultural Sciences, 334 Xueshan Road, Wenzhou 325005, China

**Keywords:** basic leucine zipper 4, cytokinin, soybean, nodule

## Abstract

Legumes have the capability to form nodules that facilitate symbiotic nitrogen fixation (SNF) with rhizobia. Given the substantial energy consumption during the process of SNF, legumes need to optimize nodule number in response to everchanging environmental scenarios. The TGACG BINDING FACTOR1/4 (TGA1/4) are key players in the basal immune response of plants. In this study, both β-glucuronidase staining and quantitative reverse transcription PCR (qRT-PCR) demonstrated that both *GmbZIP4a* and *GmbZIP4b* are inducible upon rhizobial inoculation. To investigate their roles further, we constructed *gmbzip4a*/*b* double mutants using CRISPR/Cas9 system. Nodulation assessments revealed that these double mutants displayed a reduction in the number of infection threads, which subsequently resulted in a decreased nodule number. However, the processes associated with nodule development including nodule fresh weight, structural characteristics, and nitrogenase activity, remained unaffected in the double mutants. Subsequent transcriptome analyses revealed that zeatin biosynthesis was downregulated in *gmbzip4a*/*b* mutants post rhizobial inoculation. Supporting these findings, genes associated with cytokinin (CTK) signaling pathway were upregulated in Williams 82 (Wm82), but this upregulation was not observed in the double mutants after rhizobial treatment. These results suggest that GmbZIP4a/b positively influences nodule formation by promoting the activation of the CTK signaling pathway during the early stages of nodule formation.

## 1. Introduction

Leguminous plants possess the ability to host rhizobia within specialized root structures known as nodules, which facilitate the fixation of atmospheric N2 through a process referred to as SNF [1]. In soybean, a major protein source for humans, protein accumulation is directly impacted by the process of SNF [2]. Increasing the nodule number appears to be a straightforward and effective strategy for boosting soybean yield and protein content. However, this approach negatively affects plant growth due to the high energy consumption in the process of nitrogen fixation [3]. Soybean mutants exhibiting super nodulation phenotypes are often associated with stunted shoot growth and reduced yield, primarily due to the excessive consumption of photosynthates [4,5,6]. Recent studies have revealed that the *rhizobially induced cle1a*/*2a* (*ric1a*/*2a*) mutants show increased nodule numbers, along with enhanced carbon and nitrogen acquisition, ultimately improving yield, oil content, and protein content [7]. This represents an optimal solution to the challenge of optimizing soybean nodule number, as it helps balance increased nitrogen fixation with greater carbon production. In addition to human interventions, such as CRISPR/cas9, soybean plants must also optimize nodule number to fluctuating environmental conditions. External environmental signals also influence these characteristics through systemic signaling pathways [8]. For instance, high levels of inorganic nitrogen typically suppress nodule formation and development, while also inducing senescence in mature nodules [9,10]. Key genes such as *NIN-LIKE protein* (*NLP*) and *NITRATE TRANSPORTER* (*NRT*) have been demonstrated to play significant roles in nitrate signaling and acquisition [11,12,13,14]. Despite these advances, the identification of additional regulatory factors implicated in the process of SNF continues, including *SNAP*, *NAC039*, *NAC018*, *CIN1*, *FUN*, and *ESN1*/*2* [10,15,16,17,18]. Therefore, a more comprehensive understanding of the regulatory network, which controls nodulation in soybean is still needed to refine the process and optimize its benefits.

TGACG-BINDING FACTORs were originally identified based on their capacity to bind to the activating sequence 1 (as-1 element), which comprises TGACG binding motifs [19]. In *Arabidopsis*, a total of ten TGAs have been identified, seven of which participate in basal immune responses through interactions with *nonexpresser of PR gene 1* (*NPR1*) [20]. Notably, TGA2/3/5/6/7 constitutively interact with *NPR1* in both planta and yeast systems, whereas TGA1/4 interact with *NPR1* primarily in salicylic acid (SA)-induced leaves [21]. Additionally, *tga1-1 tga4-1 npr1-1* triple mutant exhibits increased susceptibility to pathogens compared with *tga1-1 tga4-1* and *npr1-1* [22]. This indicates that the downstream target genes of TGA1/4 are not limited to *NPR1* and the biological function of TGA1/4 may also be distinct from other TGA family members. Notably, the TGA-NPR1 regulatory module is not restricted to *Arabidopsis* but has also been observed in other species, including *Oryza sativa* (rice) [23], *Triticum aestivum* (wheat) [24], and *Brachypodium distachyon* [25]. Beyond their roles in pathogen-related responses, *KD*-*hvnpr1* mutants exhibit a loss of characteristic spatiotemporal colonization patterns and reduced bacterial multiplication, suggesting a potential role for TGAs in mediating symbiotic interactions between plants and beneficial bacteria [26]. Mutual symbiosis with legumes results in a shift where plants initially activate their immune systems in response to rhizobial infection prior to recognizing the mutualistic relationship [27]. In line with that is the expression of *pathogenesis-related gene 1* (*GmPR1*), which is induced when NPR1 is upregulated in the roots following inoculation [28]. Hence, further investigation is necessary to determine whether TGA orthologues play a regulatory role in the establishment of nodulation in soybean.

Root architecture plays a critical role in regulating symbiosis traits. In *Medicago*, reduced lateral root density correlates with a significant decrease in nodule formation [29]. Similarly, deep-rooting soybean plants exhibit enhanced arbuscular mycorrhizal fungi (AMF) colonization under low phosphorus conditions [30]. The initiation of the symbiotic process is triggered by the exchange of chemical signals between the root system and rhizobia. The infection subsequently leads to the induction of root hair curling by rhizobia, facilitating the entrapment of bacterial colonies and the subsequent formation of nodules [31,32]. Overexpression of *GmEXPB2* leads to expanded root hair zones and increased root hair density, which significantly enhances both rhizobia infection and nodule formation [33]. In contrast, the knockdown of *MtFER* results in decreased root hair density, which impairs the initiation of infection threads [34]. Interestingly, *tga1 tga4* double mutants display a reduced root system, characterized by a shorter primary root length and decreased lateral root density [35]. Subsequent analyses indicated that TGA1/4 influence lateral root and root hair development by modulating the transcription levels of *NRT2.1* and *NRT2.2*, which are key regulators in nitrate transport [35,36]. Based on these findings, it is plausible to hypothesize that TGA1/4 orthologues in legumes may similarly impact nodule number by regulating root architecture.

The establishment of nodules necessitates the coordinated reinitiation of cell divisions and organogenesis. CTK plays a pivotal role in nodule organogenesis, with several components implicated in CTK signaling having been identified in the context of nodulation [37,38]. The activation of cortical cell division requires the expression of *SHORT ROOT* (*SHR*)/*SCAREROW* (*SCR*), which can be induced through the overexpression of *NIN* or by the exogenous application of CTK [39]. Isopentenyl transferases (IPTs) are key players in the initial and rate-limiting steps of CTK biosynthesis [40]. In *Lotus japonicus*, *IPT3* is shown to be required for nodule establishment and development [41,42]. Notably, the expression of *LjIPT2*/*3*/*4* responds to rhizobia inoculation, although these three CTK biosynthesis genes show distinct early and late expression patterns throughout nodule development [43]. Specifically, *IPT2* and *IPT4* contribute to the early CTK burst, and *ljipt4* single mutant shows a reduced nodule number [43]. Correspondingly, *MtIPT3*/*4*/*5* are significantly upregulated following rhizobia inoculation [44]. LONELY GUY (LOG) and cytochrome P450 monooxygenases (CYP735A1/2) are also implicated in CTK biosynthesis and nodule formation [45,46]. In *Medicago*, *MtLOG1* is predominantly localized in the dividing cells of nodule primordia. Transgenic hairy roots expressing *MtLOG1* RNAi and *35S:LOG1* constructs both demonstrate decreased nodule number [47]. Similarly, although *CYP735A* has been shown to respond to inoculation and nod factor, recent findings clarify its positive role in regulating nodule number in *Lotus japonicus* [46]. Additionally, *CYTOKININ OXIDASE*/*DEHYDROGENASE* (*CKX*) expression is also induced by nod factor during the early stages of nodule initiation [48]. Beyond nodulation, CTK also influences plant immunity against pathogens. Alterations in CTK level in plants overexpressing *CKX* or *IPT* correlate with changes in basal immunity. TGA3 has been reported to bind to the promoter of *ARR2*, establishing a connection between CTK signaling and basal defense [49]. However, the relationship between TGA1/4 or their orthologues and CTK signaling remains unclear. In this study, we observed that both *GmbZIP4a*/*b* respond to rhizobia inoculation. The absence of GmbZIP4a/b led to a significant reduction in the number of nodules per plant, although nodule development remained unaffected. Correspondingly, root hair deformation was markedly decreased in the double mutants. Transcriptional analyses indicated that mutations in *GmbZIP4a*/*b* resulted in the downregulation of genes associated with CTK biosynthesis, genes which have previously been shown to play a critical role in regulating nodule establishment.

## 2. Results

### 2.1. Transcriptional Levels of GmbZIP4a/b Were Rhizobia-Inducible

**Figure 1:**
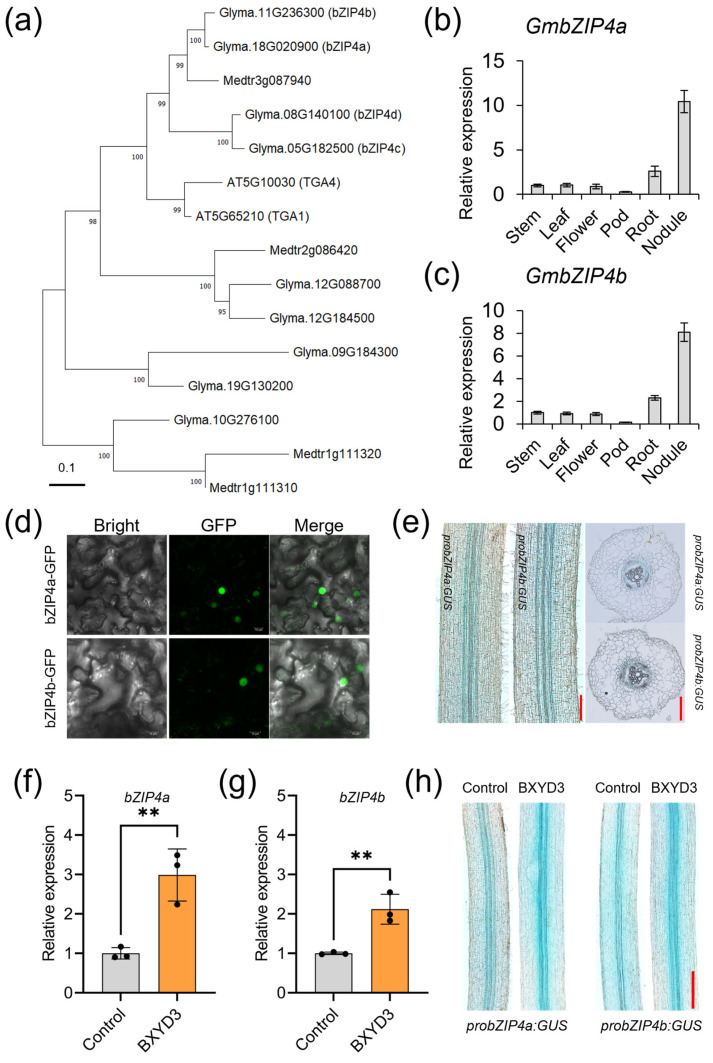
Both *GmbZIP4a* and *GmbZIP4b* exhibit responsiveness to rhizobia inoculation. (**a**) Phylogenetic analysis of the TGA1/4 orthologues in *Glycine max*. (**b**,**c**) Tissue-specific expression pattern of *GmbZIP4a* and *GmbZIP4b*. The relative expression levels of these two genes in the stem were set to 1.0. The error bar indicates standard error (SE). (**d**) Subcellular localization of GmbZIP4a/b-GFP via tobacco constitutive expression. Green fluorescence indicates nuclear localization of GmbZIP4a/b protein. Scale bar = 10 μm. (**e**) Histochemical analysis of *probZIP4a:GUS* and *probZIP4b:GUS* expression in the root under N-free condition. Scale bar = 100 μm. (**f**,**g**) *GmbZIP4a* and *GmbZIP4b* are induced by rhizobia inoculation. Each black dot represents a biological replicate. Asterisks indicate a significant difference from the control: ** *p* < 0.01 (two-way ANOVA). The error bar indicates SE. (**h**) Histochemical analysis of *probZIP4a:GUS* and *probZIP4b:GUS* expression in the root after rhizobia inoculation. Scale bar = 500 μm.

Considering the complex genomic composition of soybean, a phylogenetic analysis was conducted, revealing four orthologues of TGA1/4 designated as GmbZIP4a, GmbZIP4b, GmbZIP4c, and GmbZIP4d (Figure 1a). To investigate the tissue-specific expression profiles of these *bZIP* genes, a series of qRT-PCR assays were performed. Analysis revealed that *GmbZIP4a*/*b* are predominantly expressed in nodules and roots (Figure 1b,c). In contrast, the transcription levels of *GmbZIP4c*/*d* are specific to nodules, with their expression in roots being comparable to or lower than their expression in stems (Figure S1a,b). Consequently, this study primarily focuses on *GmbZIP4a* and *GmbZIP4b*. In line with TGA1/4, both GmbZIP4a/b exhibited nuclear localization in tobacco transient expression assay (Figure 1d). Additionally, histochemical analysis revealed that *bZIP4a*/*b* is specifically expressed in the vascular bundles of hairy roots (Figure 1e). These results suggest that bZIP4a/b in soybean share a high degree of structural and functional similarities with TGA1/4. To assess whether GmbZIP4a/b are involved in the process of nodule formation, qRT-PCR assays were performed with inoculated roots. Both *bZIP4a*/*b* were significantly upregulated at 4 days post inoculation (dpi) (Figure 1f,g). Furthermore, hairy roots containing *probZIP4a/b: GUS* vectors were inoculated with rhizobia. As shown in the images, the inoculated hairy roots have darker blue vascular tissues than controls at 4 dpi (Figure 1h), suggesting that *GmbZIP4a*/*b* are rhizobia-inducible at the transcriptional level. Therefore, these two transcription factors may participate in the regulatory network that governs the nodulation process in soybean. However, the underlying mechanisms remain to be fully elucidated.

### 2.2. Generation of Gmbzip4a/b Double Mutants Using CRISPR/Cas9

**Figure 2:**
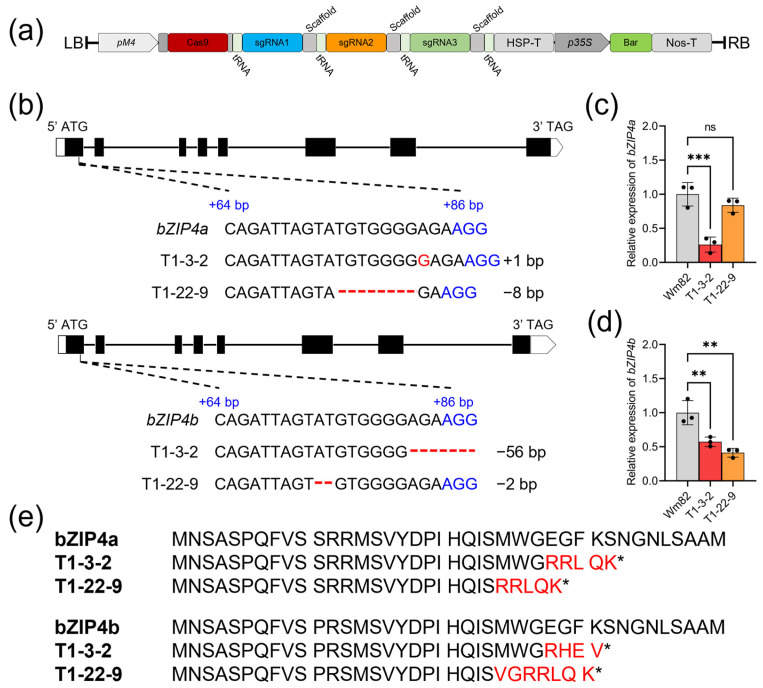
Construction of *gmbzip4a*/*b* double mutants. (**a**) Schematic plot of the pGES401 vector, which contains sgRNA fragments designed for the editing of *bZIP4a*/*b*. (**b**) Mutation types of two different homozygotes of *gmbzip4a*/*b* double mutants. (**c**,**d**) Relative expression of *bZIP4a* and *bZIP4b* in two homozygote double mutants. Asterisks indicate significant differences compared with Wm82: ** *p* < 0.01; *** *p* < 0.001; ns, not significant (two-way ANOVA). The error bar indicates SE. (**e**) Predicted amino acid sequence generated by SnapGene, illustrating the amino acid sequence from the start codon to the termination site (*) resulting from the mutations. Amino acids marked in red indicate potential sequence changes caused by mutations. Asterisks indicate termination sites.

To elucidate the role of GmbZIP4a/b in the process of SNF, a genome-editing vector pGES401, previously reported in [50], was engineered to simultaneously target and modify both genes (Figure 2a). Two stable and heritable *gmbzip4a*/*b* double mutants were obtained in the T2 generation. Specifically, the T1-3-2 line harbored a single base insertion in *bZIP4a* and a 56-base deletion in *bZIP4b*, whereas the T1-22-9 line exhibited an eight-base deletion in *bZIP4a* and a two-base deletion in *bZIP4b* (Figure 2b). The sequencing results of these three sgRNAs in the two double mutants can be found in Supplementary Data (Figure S2). Both *bZIP4a* and *bZIP4b* were downregulated in the T1-3-2 line (Figure 2c,d). In the T1-22-9 line, the expression of *bZIP4a* was only slightly, and statistically insignificantly, decreased (Figure 2c), while *bZIP4b* was strongly suppressed (Figure 2d). Predictions of the potential amino acid sequence alterations caused by the eight-base deletion in T1-22-9, performed using SnapGene, suggested that both protein sequences experienced premature termination in these two lines (Figure 2e). Consequently, the T1-22-9 line is expected to exhibit phenotypic changes to those of the T1-3-2 line. This raises an intriguing question: what phenotypes will the loss of bZIP4a/b induce in these two mutant lines?

Before investigating nodulation phenotypes, we assessed the overall plant phenotypes of the *gmbzip4a*/*b* double mutants, including T1-3-2, at 25 dpi under nitrogen-free conditions. The double mutants were observed to have reduced plant height and lower SPAD values (Figure S3a–c), indicating potential impairment in nitrogen acquisition. Since soybean plants acquire nitrogen through both root systems and nodules, further research is needed to pinpoint the specific step at which bZIP4a/b are involved. Subsequently, we conducted a hydroponic assay to further investigate plant phenotypes in the absence of inoculation, under 0.1 and 1 N conditions. After three weeks of hydroponic cultivation, no significant differences were observed between the double mutant and Wm82 control in multiple traits, including plant height, root area, and dry weight at either nitrogen levels (Figure S3d–i). These results suggest that the observed differences between the *bzip4a*/*b* double mutants and Wm82 are primarily due to SNF, rather than general plant growth traits.

### 2.3. Gmbzip4a/b Double Mutants Showed Decreased Nodule Numbers

**Figure 3:**
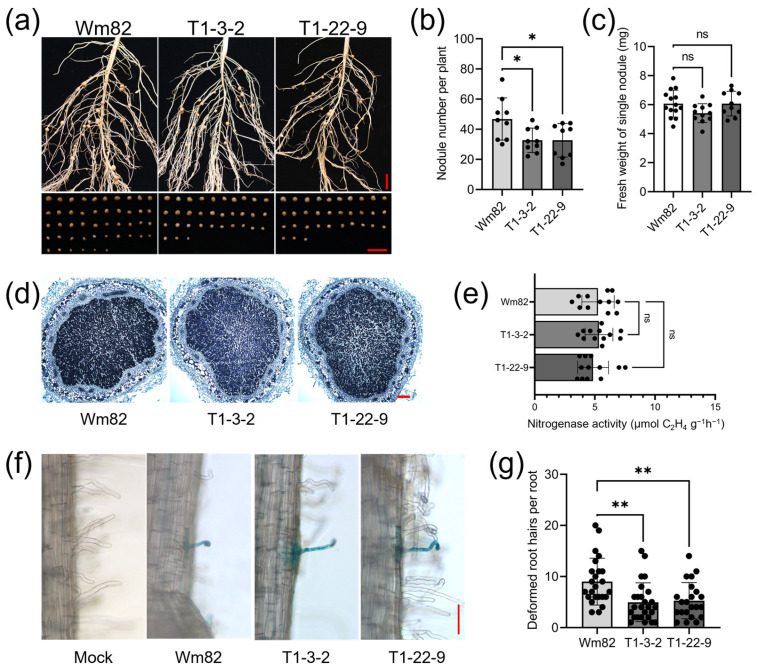
GmbZIP4a/b were involved in regulating nodule number in soybean. (**a**) The *gmbzip4a*/*b* double mutants showed reduced nodule number. Scale bar = 1 cm. (**b**,**c**) Nodule number and fresh weight of single nodules of *gmbzip4a*/*b* and WT (*n* ≥ 9, each black dot represents an individual seedling). Asterisk indicates a significant difference from Wm82: * *p* < 0.05; ns, not significant (two-way ANOVA). The error bar indicates SE. (**d**) Nodule sections of double mutants under N-free conditions. Scale bar = 200 μm. (**e**) Nitrogenase activity of single nodules of *gmbzip4a*/*b* and WT under N-free conditions (*n* ≥ 12, each black dot represents an individual nitrogenase activity value); ns, not significant (two-way ANOVA). The error bar indicates SE. (**f**) Pictures of deformed root hairs, which were stained blue. Uninoculated root hairs were used as a negative control. Scale bar = 100 μm. (**g**) Deformed root hair number of each root fragment (*n* ≥ 20, each black dot represents an individual lateral root). The asterisks indicate a significant difference from Wm82: ** *p* < 0.01; (two-way ANOVA). Error bar indicates SE.

To investigate the nodulation phenotypes of *gmbzip4a*/*b* double mutants, we inoculated these two lines with BXYD3 under N-free conditions. Nodule counts were conducted and analyzed at 14 dpi. The results indicated that both T1-3-2 and T1-22-9 exhibited a significantly decreased nodule number (Figure 3a,b). However, no changes were observed in the fresh weight of a single nodule in either line (Figure 3c). This result suggests that GmbZIP4a/b specifically influence nodule number rather than nodule development. To further verify this hypothesis, we subsequently performed paraffin sectioning of the nodules. Compared with Wm82, no obvious morphological alterations were found in these two double mutants (Figure 3d). Nitrogenase activity, an important indicator for nodule development and nitrogen-fixing capacity, was then assessed in mature nodules collected from T1-3-2 and T1-22-9. As anticipated, the nitrogenase activities of the two double mutants did not differ from those of Wm82 (Figure 3e). These results support the conclusion that the losses of bZIP4a/b do not impact the developmental processes of the nodules.

Following the completion of mutual recognition between the root hairs and rhizobia, rhizobia penetrated the root system through infection threads, accompanied by the curling of the root hairs. To quantify the infection events of *gmbzip4a*/*b*, we inoculated 7-day-old seedlings of T1-3-2 and T1-22-9 with USDA110-GUS, which contains a β-glucuronidase tag. The number of deformed root hairs was counted and analyzed at 7 dpi. Both double mutants demonstrated a significant reduction in deformed root hairs (Figure 3f,g). These findings suggest that the decreased nodule number observed in these mutants can be attributed to a lower frequency of infection events.

### 2.4. The Impaired Nodule Establishment in Gmbzip4a/b Is Associated with Zeatin Biosynthesis

**Figure 4:**
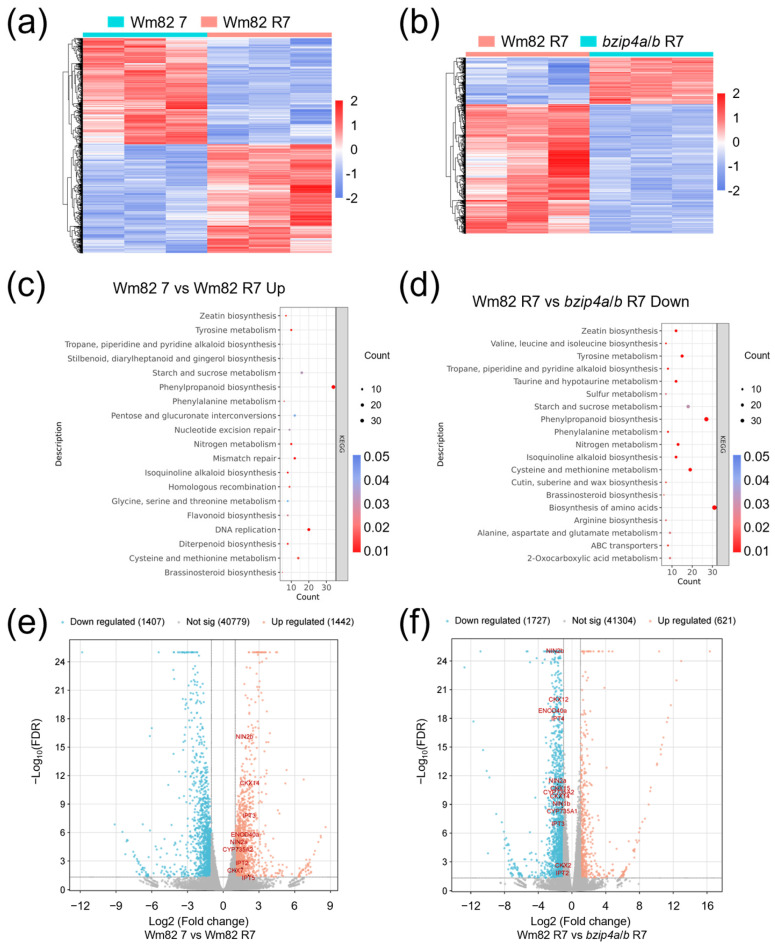
Zeatin biosynthesis was associated with reduced nodule establishment. Panels (**a**,**b**) present heat maps comparing Wm82 7 to Wm82 R7, as well as Wm82 R7 to *bzip4a*/*b* R7. Panels (**c**,**d**) provide a KEGG analysis of the upregulated genes in Wm82 R7, and the downregulated genes in *bzip4a*/*b* R7, respectively. Finally, panels (**e**,**f**) depict volcano plots for the DEGs between Wm82 and Wm82 R7 and Wm82 R7 and *bzip4a*/*b* R7, respectively.

To gain a deeper understanding of the transcriptional reprogramming occurring during nodule establishment, we collected root samples at 7 dpi. At the transcriptional level, significant differences were observed in Wm82 before and after rhizobia inoculation, as well as between the mutant and wild types under the same conditions (Figure 4a,b). All DEGs can be found in Supplementary Table S2. Considering that TGA1/4 typically function as transcriptional activators, our analysis primarily focused on the downregulated genes in *bzip4a*/*b* R7 and upregulated genes in Wm82 R7. Notably, zeatin biosynthesis was significantly enriched in both data sets (Figure 4c,d). The complete KEGG analysis results are available in Supplementary Table S3. Further analysis revealed that the zeatin biosynthesis-associated genes, including *GmIPT2*, *GmIPT3*, and *GmIPT5*, *GmCYP735A1*, *GmCYP735A2*, as well as *CKX7* and *CKX14*, were significantly induced in Wm82 post inoculation (Figure 4e). In contrast, zeatin-related genes such as *GmIPT2*, *GmIPT3*, *GmIPT4*, *GmCYP735A1*, *GmCYP735A2*, *GmCKX2*, *GmCKX12*, *GmCKX14*, and *GmCKX15* exhibited suppressed expression levels in T1-3-2 compared with Wm82 after inoculation (Figure 4f). These results indicate a potential role of a zeatin biosynthesis pathway in the phenotypic differences in nodule number between gmbzip4a/b and Wm82.

The expression levels of *GmNIN*s, which are key indicators of the rhizobial infection process, were also examined. At 7 dpi, *GmNIN2a*, *GmNIN2b*, and *GmENOD40a* were found to be upregulated in Wm82 following inoculation (Figure 4e). Conversely, *GmNIN1b*, *GmNIN2a*, *GmNIN2b*, and *GmENOD40a* exhibited downregulation in T1-3-2 at the same time point (Figure 4f). Collectively, these findings suggest a potential linkage between the nodulation traits observed in the *gmbzip4a*/*b* double mutants and the zeatin signaling pathway.

### 2.5. Zeatin Biosynthesis Genes Were Not Rhizobia-Responsive in Gmbzip4a/b Mutants

**Figure 5:**
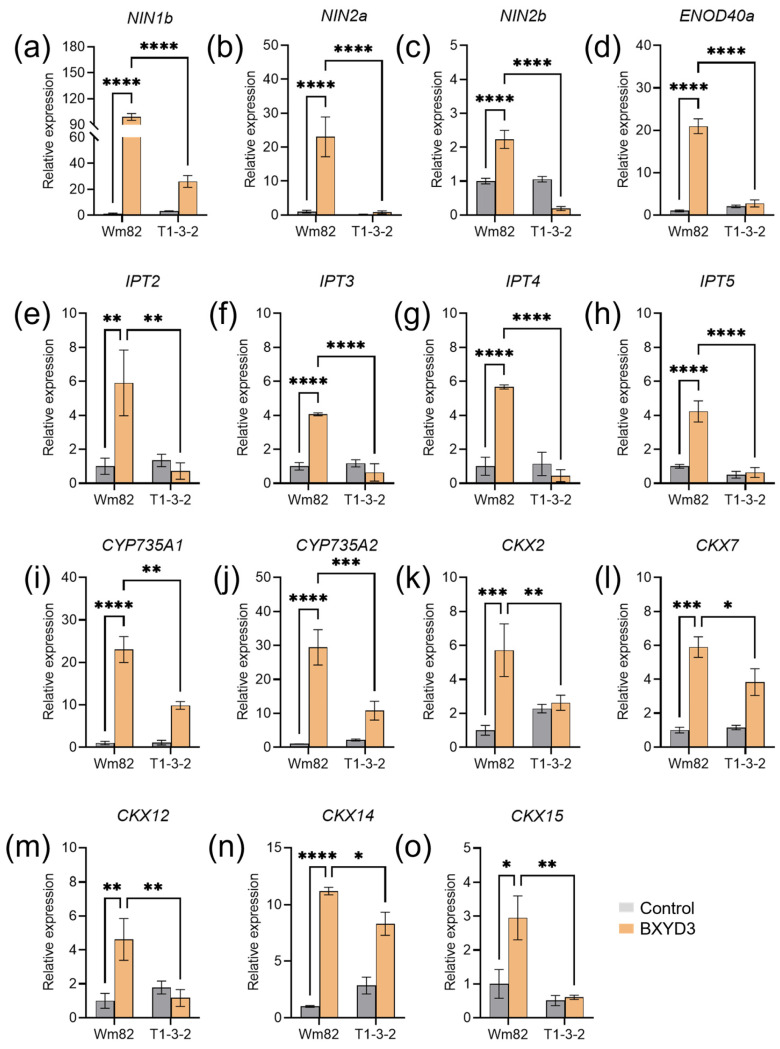
A qRT-PCR analysis of nodulation-related DEGs. (**a**–**d**) Marker genes of nodulation during the early stages post inoculation. (**e**–**j**) Genes that are involved in CTK biosynthesis process. (**k**–**o**) CTK metabolism-related genes. The 2^−ΔΔCT^ method was used for relative expression analysis. Three biological replicates were included. Asterisks indicate a significant difference from the control: * *p* < 0.05, ** *p* < 0.01, *** *p* < 0.001, **** *p* < 0.0001 (two-way ANOVA). The error bar indicates SE.

To verify the findings related to early nodulation response genes observed in the transcriptome data, we conducted a series of qRT-PCR experiments. Consistent with the transcriptome data, *GmNIN1b*, *GmNIN2a*, *GmNIN2b*, and *GmENOD40a* were all upregulated in Wm82 at 7 dpi, but not in T1-3-2 (Figure 5a–d). Four *IPT* genes, specifically *GmIPT2*, *GmIPT3*, *GmIPT4*, and *GmIPT5*, were not upregulated in *gmbzip4a*/*b* double mutants at 7 dpi (Figure 5e–h). Additionally, *CYP735A1* and *CYP735A2*, which are involved in zeatin biosynthesis, exhibited similar patterns; both were induced in Wm82 post inoculation but remained uninduced in the *gmbzip4a*/*b* mutants (Figure 5i,j).

On the other hand, several *CKX* genes exhibited opposite expression trends between T1-3-2 and Wm82 at 7 dpi. While CKXs are known for their roles in the irreversible degradation of CTK, they also play a critical role in regulating CTK homeostasis, which is essential for nodule formation [48,51]. Therefore, we further examined the expression of these *CKX* genes, as these five *CKX* genes showed decreased expression in T1-3-2 after inoculation compared with those in Wm82 (Figure 5k–o). These results suggest that CTK levels must be tightly regulated to support effective nodule formation, as excessively high CTK concentrations can be detrimental to this process. CKXs likely play a critical role in maintaining optimal CTK levels for nodulation.

## 3. Discussion

The establishment of nodules is a complex process that requires a successful interaction between rhizobia and host plants, leading to rhizobial infection and subsequent nodule organogenesis. However, symbiotic nitrogen fixation in plants is an energy-intensive process [52] and plants need to moderate nodule formation through the AON pathway to optimize nitrogen acquisition. This regulation ensures that excessive nodule formation does not negatively impact plant yield [7,53]. In a recent study, Zhong et al. created a series of AON genes-related mutants using a multiplexed CRISPR/Cas9 mutagenesis system. Phenotypic analysis of nodulation revealed that excessive nodule numbers in *nark* and *ric1a*/*1b*/*2a*/*2b nic1*/*2* (designated *ric-6m*) reduced shoot growth [7]. In contrast, *ric1a*/*2a* and *ric1b*/*2b* exhibit moderately increased nodule numbers, which lead to increased nitrogen and chlorophyll content, as well as a 10–31% grain yield increase [7]. This experimental evidence suggests that optimizing nodule number can enhance crop productivity by balancing the source–sink relationship between nitrogen and carbon assimilation [6]. Furthermore, knockout of *GmNLP1*/*4* confers notable nitrate-tolerant nodulation phenotypes in soybean, including a smaller reduction in infection thread and nodule number, as well as unaffected nitrogenase activity upon nitrate treatment [54]. Additional genes that contribute to nodule symbiosis beyond typical AON-related components, including *PSK-δ*, *VPT*s, and *SymCEP7*, have recently been identified [55,56,57]. The application of PSK-δ and SymCEP7 peptides have been shown to significantly increase nodule number in *Medicago*, *Trifolium repens*, and *Lotus japonicus*, respectively [55]. However, mutants of *vpt2* and *vpt3* demonstrate significantly reduced nodule formation under various phosphate conditions [56]. In our study, we identified GmbZIP4a/b, orthologues of AtTGA1/4, as positive regulators of nodule formation in soybean. These two transcription factors appear to be involved in the regulatory networks that control nodule formation in soybean. These newly found genes are not included in the typical AON pathway, implying that the molecular mechanism governing nodule formation in legumes remains to be fully elucidated. Furthermore, how to effectively utilize these genes involved in regulating the number of nodules remains a challenge that still needs to be addressed. 

Root traits are crucial for crop performance, particularly in scenarios where soil nutrients are limited. Consequently, modifying root architecture represents a significant strategy for crop adaptation to nutrient deficiency conditions. In *Arabidopsis*, double mutants of *tga1 tga4* exhibit impaired lateral root initiation and reduced root hair density [35,36,58], indicating that the orthologues of these transcription factors in soybean may play critical roles in regulating root architecture. However, after three weeks of hydroponic treatment under 0.1 or 1 N conditions without inoculation, the T1-3-2 plants did not show significant differences in plant morphology, height, root or shoot dry weight, or root area when compared with Wm82 (Figure S3d–i). These findings suggest that functional divergence may have occurred among these orthologues. Alternatively, there could be functional redundancy among the bZIP4 family members, meaning that the double mutation of *bZIP4a*/*b* may not be sufficient to induce noticeable changes in root-related phenotypes. We concluded that the observed phenotypic differences between *gmbzip4a*/*b* and Wm82 plants were likely due to variations in their different SNF capabilities. Consistently, both double mutants exhibited a significant reduction in the number of deformed root hairs (Figure 3f,g), which subsequently led to a decrease in nodule formation (Figure 3a,b). However, analyses of the fresh weight of individual nodules, paraffin sections, and nitrogenase activity revealed no significant differences between double mutants and Wm82 (Figure 3c–e). These findings suggest that GmbZIP4a/b play positive roles in the early stages of symbiotic establishment between soybean and rhizobia but do not affect the later stages of nodule development. This insight into the functional roles of GmbZIP4a/b contributes to a deeper understanding of these two transcription factors involved in the symbiotic relationship.

Since the onset of infection, nodule establishment is significantly influenced by the homeostasis of locally produced phytohormones, particularly CTK [59]. CTK plays a crucial role in promoting the dedifferentiation of cortical cells, a process essential for initiating the early stages of nodule development [60,61]. The biosynthesis of CTK requires the action of three key enzymes. First, isopentenyl transferases (IPTs) regulate a rate-limiting step in CTKs biosynthesis, primarily producing isopentenyl adenine (iP)- and trans-zeatin (tZ)-type CTKs [40,62]. Next, cytochrome P450 enzymes, specifically CYP735A, facilitate the conversion of iP nucleotides to tZ nucleotides [43,63]. Finally, the LONELY GUYs (LOG) enzymes catalyze the conversion of cytokinin nucleotides to their active forms by cleaving the nucleotide precursors [45]. Knockdown of *IPT3* sharply reduces the endogenous CTK content, leading to fewer infection threads and nodules, along with impaired nitrogenase activity [41]. Interestingly, a grafting assay revealed that the *ipt3* in the shoots can increase nodule numbers, whereas overexpression of *LjIPT3* in shoots leads to a decreased nodule number [42]. In *Medicago*, *ipt3* mutants also display a significantly reduced nodule number [59]. These studies highlight the complexity of the regulatory mechanisms governing nodule formation, where the levels and context of CTK signaling are orchestrated by IPTs. The synthesis of *trans*-Zeatin by CYP735A is promoted during nodulation, but *cyp735A* mutants show unchanged nodulation in *L. japonicus* [43]. Following studies revealed that *cyp735A* mutants show a sharply decreased nodule number until three weeks post inoculation with IRBG74, which infects roots in an intercellular manner [46]. Considering the transcriptome results of *CYP735A1* and *CYP735A2* in soybean at 7 dpi (Figure 4e,f), we believe these two genes may play important roles during nodulation in soybean. In *Medicago*, LOG1 is reported to function in a CRE1-dependent manner in dividing cells of the nodule primordium. Significant reductions in nodule number are observed in *MtLOG1* RNAi roots [47]. Similarly, overexpression of *LOG1* also results in a dramatically impaired nodule number in the transgenic roots [47]. These observations confirm that *LOG*s are critical regulators of nodulation process due to their ability to convert CTKs to active forms. However, it is noteworthy that no *LOG* genes were found in the transcriptome data in our study. This suggests that while bZIP4 regulates nodule number, LOGs do not appear to be involved in this process. 

**Figure 6:**
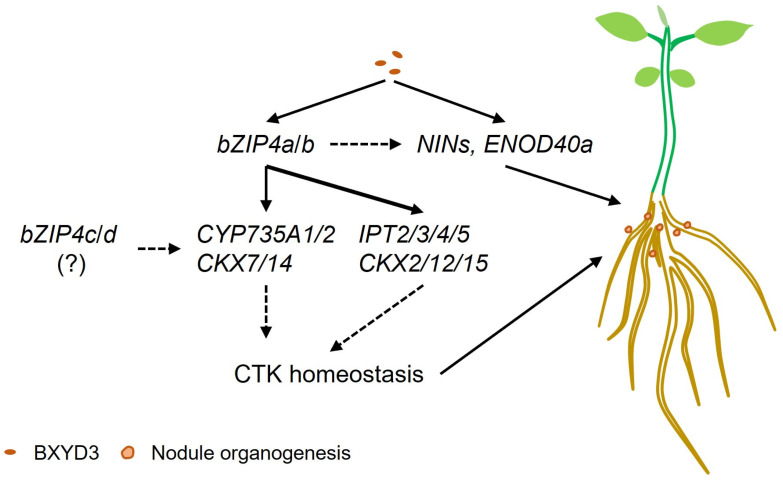
Proposed model of GmbZIP4a/b positively regulating nodule number in soybean. Rhizobial infection induces the expression of *NIN*s and *ENOD40a*, key nodulation-responsive genes, while simultaneously upregulating *GmbZIP4a*/*b*. GmbZIP4a/b promote the expression of CTK-related genes, including *IPT*s, *CYP735A*s, and *CKX*s, thereby contributing to the regulation of CTK homeostasis in roots. During this process, bZIP4c/d may be involved in the regulation of *CYP735A1*/*2* and *CKX7*/*14*. In contrast, the absence of GmbZIP4a/b may disrupt CTK homeostasis, leading to a decreased nodule number.

Excessive phytohormones are also detrimental to the establishment of nodules. CKXs are responsible for irreversible degradation of CTK, thus playing a critical role in regulating CTK homeostasis [51]. The mRNA level of *LjCKX3* is induced by nod factor during the early stage of nodule initiation. Phenotypic analysis has revealed that *ckx3* mutants have significantly decreased infection threads and nodule number [48]. In our findings, the relative expression of *CKX7*/*14* were induced in Wm82 at 7 dpi while those of *CKX2*/*12*/*14*/*15* were downregulated in T1-3-2 (Figure 4e,f). These findings were further corroborated by qPCR data (Figure 5k–o), demonstrating that CKXs tightly regulate CTK accumulation during nodulation. Recent research shows that higher CKX6 activity in roots does not alter root nodulation and SNF in Chickpea (*Cicer arietinum* L.) [64], suggesting that CKXs may have function variations in nodule formation across different leguminous plants. Furthermore, we noticed that while the expression of *IPT*s and *CKX2*/*12*/*15* failed to be induced in T1-3-2 at 7 dpi, the expression of *CYP735A1*/*2* and *CKX7*/*14* remained induced in the double mutants. These findings suggest that *CYP735A1*/*2* and *CKX7*/*14* may be regulated by additional factors, such as GmbZIP4c/d. Overall, this study demonstrates that GmbZIP4a/b positively regulate nodulation due to the rhizobia-induced local zeatin biosynthesis and metabolism. Furthermore, the expression of *CKX*s were induced after inoculation to maintain the CTK homeostasis. In contrast, insufficient induction of zeatin biosynthesis-related genes and *CKX*s might lead to inappropriate CTK levels, which disrupt nodule initiation in the double mutants. In summary, we propose that rhizobial infection not only upregulates the expression of nodulation-responsive genes such as *NIN*s and *ENOD40a* but also enhances the transcriptional levels of *bZIP4a*/*b* (Figure 6). Furthermore, bZIP4a/b promote the expression of genes like *IPT*s, *CYP735A*s, and *CKX*s. This regulatory mechanism may influence the CTK homeostasis in roots, thereby positively regulating the number of nodules in soybean roots.

## 4. Materials and Methods

### 4.1. Plant Growth Conditions

The soybean (*Glycine max*) cultivar Williams 82 was used throughout the study, and inoculation was performed with *Bradyrhizobium elkanii* strain BXYD3 [65]. Sterilized seeds were geminated in vermiculite, and we inoculated the seedlings with BXYD3 suspension (OD = 0.08) at seven days post germination. The plants were treated with N-free solution containing MgCl_2_ 2.5 μM, MgSO_4_ 0.5 mM, K_2_SO_4_ 1mM, MnSO_4_ 0.5 μM, ZnSO_4_ 1.5 μM, CuSO_4_ 0.5 μM, (NH_4_)·Mo_7_O_24_ 0.15 μM, KH_2_PO_4_ 0.25 mM, NaB_4_O_7_ 0.25 μM, Fe-Na-EDTA 40 μM, and CaCl_2_ 1.2 mM. For 1N media, Ca (NO_3_)_2_ 0.6 mM, (NH_4_)_2_SO_4_ 0.25 mM, KNO_3_ 0.95 mM were used. The amount of these three components was reduced to 1/10 for 0.1N media. Nodule phenotypes including nodule number, fresh weight, and paraffin sections were investigated at 14 dpi. For nitrogenase activity measurement, mature nodules were collected at 25 dpi. To investigate the tissue-specific expression pattern of *bZIP4*s, lateral roots, stem fragments, leaves, nodules flowers, and young pods were collected. For the marker genes’ expression analysis, the roots were harvested at 0, 4, and 7 dpi; non-inoculated roots were used as a negative control. All tissues were then used for RNA extraction and qRT-PCR analysis.

### 4.2. RNA Extraction and qRT-PCR Analysis

Total RNA was isolated using FastPure Universal Plant Total RNA Isolation Kit (RC411-01, Vazyme, Nanjing, China) according to the manufacturer’s instructions. The reverse transcription was performed with PrimeScriptTM RT reagent Kit (RR037Q, TAKARA, Osaka, Japan). The qRT-PCR was carried out on three biological repeats with LightCycler 96 (Roche, San Francisco, CA, USA) using AceQ qPCR SYBR Green Master Mix (Q111-02, Vazyme, Nanjing, China) in 20 μL reaction volumes (2× SYBR mix, 10 µL; primer-F, 0.6 µL; primer-R, 0.6 µL; H_2_O, 6.8 µL; template cDNA, 2 µL). *TefS1* was used as a reference gene to calculate relative expression levels using the 2^−ΔΔCT^ method [66]. Specific primer sequences are listed in Supplementary Table S1.

### 4.3. Transient Expression of bZIP4a/b CDS Sequence in Nicotiana benthamiana

Transient expression in *Nicotiana benthamiana* is usually used to determine the subcellular location of a protein when tagged with a reporter such as GFP. We constructed pGWB605-bZIP4a and pGWB605-bZIP4b, utilizing the pGWB605 backbone with GFP as a reporter to visualize the localization of these transcription factors within the cells. The design and cloning of bZIP4a and bZIP4b coding sequences were performed using specific primers outlined in Supplementary Table S1. The transient expression assay was conducted following established protocols as previously described [67].

### 4.4. Histochemical GUS Staining of Tissue Sections

To figure out the expression pattern of *bZIP4a*/*b* in roots, a 2000 bp fragment upstream of the ATG start codon of *GmbZIP4a*/*b* was designated as the promoter region. The pUbi vector, which includes a GUS (β-glucuronidase) reporter gene, was first digested with *BamH*I and *Pst*I to create suitable cloning sites. Subsequently, the cloned promoter fragments were fused to the GUS tag via Gibson assembly. Transgenic hairy roots harboring *probZIP4a:GUS* or *probZIP4b:GUS* were generated via the hypocotyl infection method as described before [68]. For the rhizobia infection response, BXYD3 infected roots were collected at 4 dpi. Root segments in GUS staining solution containing 50 mM Pi-buffered saline (Na_2_HPO_4_-NaH_2_PO_4_ buffer), 0.1% (*v*/*v*) Triton X-100, 2 mM K_3_Fe(CN)_6_, 2mM K_4_[Fe(CN)_6_]·3H_2_O, 10 mM EDTA-2Na, and 2 mM 5-bromo-4-chloro-3-indolyl-β-D-GlcA were incubated at 37 °C for one hour. The stained roots were embedded with cryogen and sectioned longitudinally and transversely to 40 μm with cryostat microtome (CM1950, Leica, DEU, Wetzlar, Germany) for GUS activity observation. Pictures were taken under a Zissis AXIO Imager. A2 microscopy (Zeiss, DEU, Oberkochen, Germany).

### 4.5. Generation of Gmbzip4a/b Double Mutants via CRISPR-Cas9

To generate heritable *gmbzip4a*/*b* double mutants through CRISPR-Cas9, three sgRNAs targeting different regions of *GmbZIP4a*/*b* were designed and cloned into pGES401 vector. Sequences of sgRNAs are listed in Supplementary Table S1. The vector was then transformed into *A. tumefaciens* strains GV3101, which was then used to infect Wm82 via *Agrobacterium*-mediated transformation. 

### 4.6. Nodule and Infection Thread Traits Analysis

For the infection thread and nodule traits observation, seeds of two homozygote double mutants, T1-3-2 and T1-22-9, were surface sterilized and sown in sterilized vermiculite. Seven-day-old seedlings were then inoculated with BXYD3 (OD600 = 0.08). Nodulation phenotypes including nodule number and fresh weight were investigated at 14 dpi. Representative nodules of each genotype were embedded with paraffin and sectioned for nodule structure investigation. Mature nodules were harvested for nitrogenase activities measurement at 25 dpi as previously described [10]. To better quantify how the absence of GmbZIP4a/b affects nodule number, a rhizobia strain *B. japonicum* USDA110 was recruited [69]. The 7-day-old seedlings were inoculated with a suspension of USDA110 (OD600 = 0.08). To investigate the infection events, the roots were cut into 5 cm fragments at the infection zone and stained with GUS. Both nodule sections and root hair deformation were all observed with a light microscope (AXIO Imager. A2).

### 4.7. Transcriptome Analysis

T1-3-2 (referred to as *bzip4a*/*b*) and Wm82 were used for the transcriptome analysis. Total RNA samples were used as input for transcriptome deep sequencing. Sequencing libraries were prepared using the NEBNext Ultra™ RNA Library Prep Kit (E7530, NEB, Ipswich, MA, USA) according to the manufacturer’s instructions. Libraries were sequenced on an Illumina NovaSeq platform (Illumina, San Diego, CA, USA) using PE150 strategy. Quality control of the raw RNA-seq reads, including adapter trimming and removal of low-quality reads, was performed using Trimmomatic v0.39 [70]. The clean reads were aligned to the cultivated soybean Wm82 v.4 refrence genome using HISAT2 v2.1.0 [71]. The number of reads mapping to each gene and the normalized expression value (FPKM) were calculated using StringTie v.1.3.6 [72]. Read count was used to perform differential-expression analysis using DESeq2 v1.3.4 with a false discovery rate < 0.05 and |log_2_ (fold change)|  ≥  1 between the treatment and control groups [73]. The online platform KOBAS v3.0 was applied to operate the KEGG enrichment analysis [74].

## 5. Conclusions

This study demonstrates that GmbZIP4a/b play crucial roles in promoting nodule formation in soybeans by activating the CTK signaling pathway during early nodulation stages. While the *gmbzip4a*/*b* double mutants exhibited reduced infection threads and nodule numbers, other nodule development processes remained unaffected. These findings highlight the importance of zeatin biosynthesis and CTK signaling in regulating effective nodulation.

## Data Availability

The data presented in this study are available in the Supplementary Materials.

## Supplementary Materials

The following supporting information can be downloaded at: https://www.mdpi.com/article/10.3390/ijms252413311/s1.
