# Supplementary material for: GmbZIP4a/b Positively Regulate Nodule Number by Affecting Cytokinin Biosynthesis in Glycine max
Source: Int J Mol Sci. 2024 Dec 11;25(24):13311. doi: 10.3390/ijms252413311 (PMC11678618; doi:10.3390/ijms252413311)
Supplement: Supplementary file 1 [file ijms-25-13311-s001.zip › Supplementary data.pdf]

Figure S1

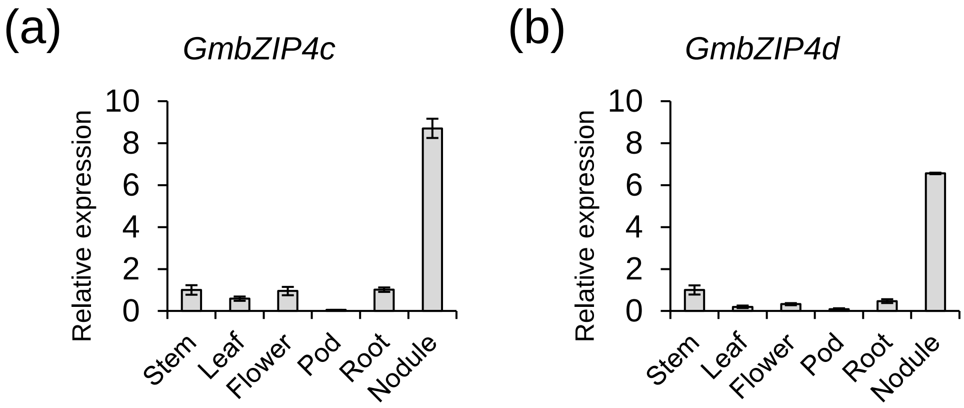

Figure S1. The tissue-specific expression of *GmbZIP4c*/*d*. (a-b) The tissue-specific expression pattern of *GmbZIP4c* and *GmbZIP4d*. The relative expression levels of these two genes in the stem were set to 1.0. The error bar indicates SE.

Figure S2

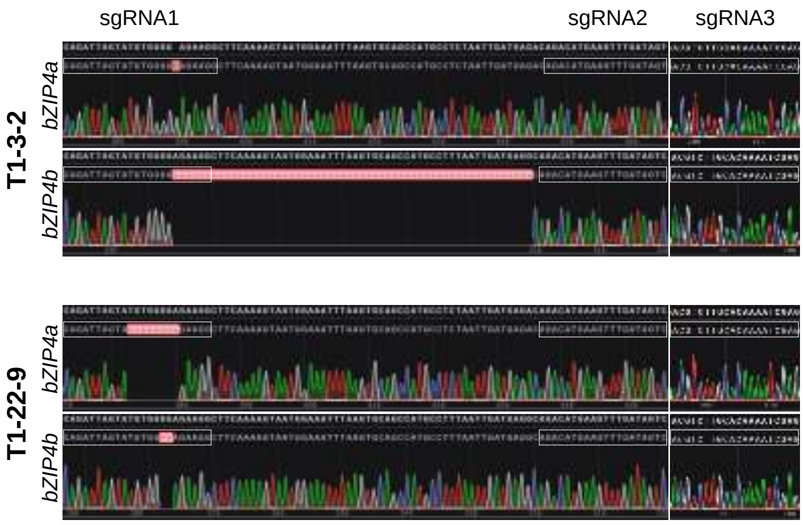

Figure S2. Sequencing results of *gmbzip4a/b* double mutants. The sequencing data of three sgRNAs in T1-3-2 and T1-22-9 respectively. The sequence of each sgRNA was labeled with white-edged transparent rectangular boxes.

Figure S3

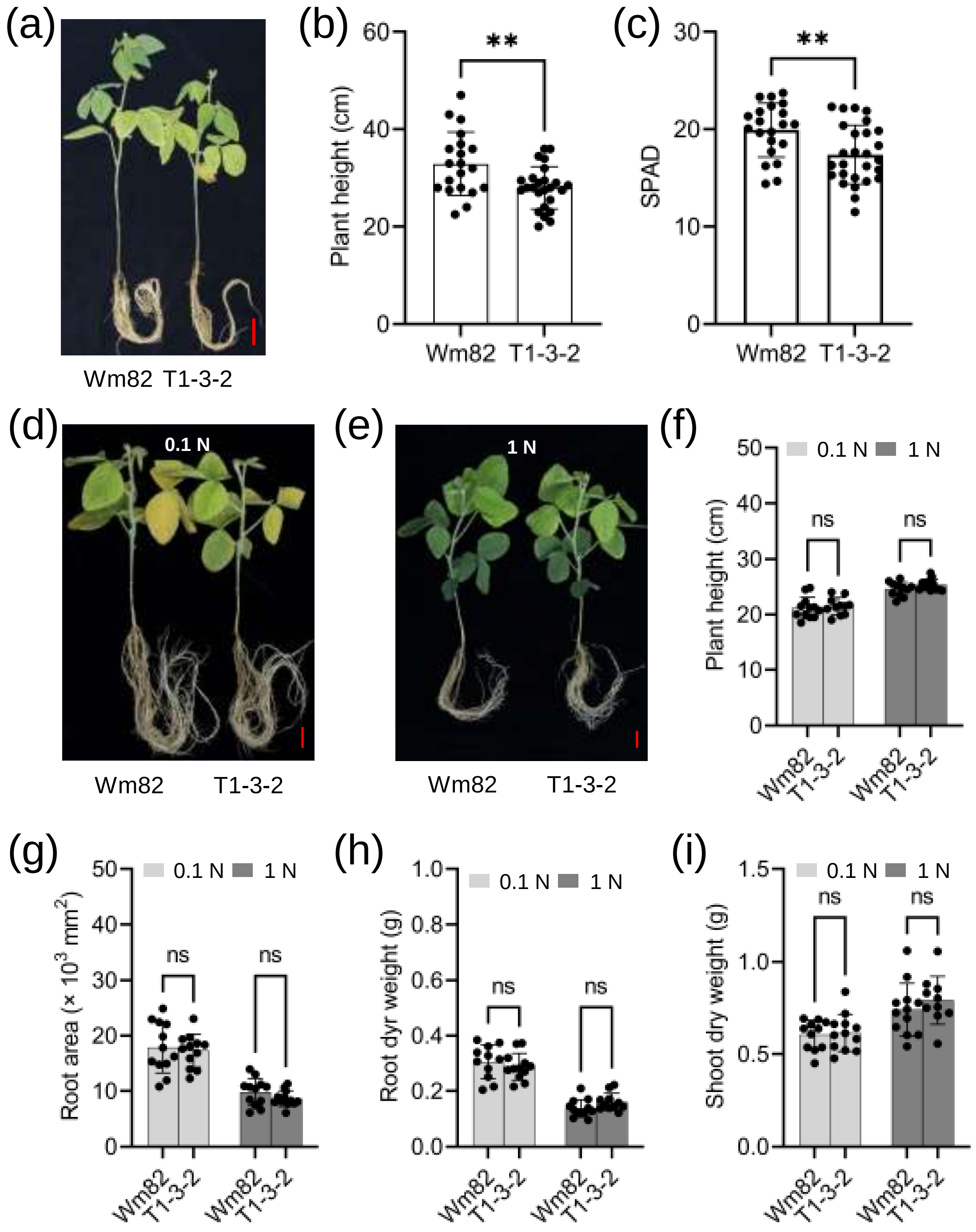

Figure S3. *gmbzip4a/b* double mutants showed smaller plants after inoculation.
(a) T1-3-2 and Wm82 plants under 0.1 N condition at 21 dpi. Scale bar = 5 cm. (b-c) Plant height and SPAD value of T1-3-2 and Wm82 under 0.1 N condition at 21 dpi (n ≥ 20, each black dot represents an individual seedling). $^{**}P < 0.01$; (two-way $_{\text{ANOVA}}$). The error bar indicates SE. (d-e) T1-3-2 and Wm82 plants under 0.1/1 N treatment. Scale bar = 5 cm. (f-i) Investigation of plant height, root area ,and dry weight of root and shoots under 0.1/1 N treatment (n ≥ 10, each black dot represents an individual seedling). ns, not significant (two-way $_{\text{ANOVA}}$). The error bar indicates SE.
